# The Efficacy of Antihypertensive Drugs in Lowering Blood Pressure and Cardiovascular Events in the Elderly Population: A Systematic Review and Meta-Analysis

**DOI:** 10.7759/cureus.52053

**Published:** 2024-01-10

**Authors:** Raheel Chaudhry, Yusuf A Siddique, Ahmad Sebai, Mustafa M Moazam, Ghazala S Virk, Yonas Tamene, Mohamed Hassouba

**Affiliations:** 1 Medicine, Baylor College of Medicine, Houston, USA; 2 Medicine, St. George's University School of Medicine, St. George's, GRD; 3 Medicine, California University of Science and Medicine, Colton, USA; 4 Psychiatry, Texas Tech University Health Sciences Center El Paso, El Paso, USA; 5 Internal Medicine, Avalon University School of Medicine, Youngstown, USA; 6 Internal Medicine, California Institute of Behavioral Neurosciences and Psychology, Fairfield, USA; 7 Pediatrics, State University of New York (SUNY) Downstate Health and Sciences University, Brooklyn, USA

**Keywords:** blood pressure-lowering medication, antihypertensive drugs, major adverse cardiovascular events, cardiovascular adverse events, hypertension

## Abstract

Cardiovascular disease is the leading cause of mortality and morbidity worldwide. One of the main risk factors for cardiovascular events is hypertension. The use of antihypertensive drugs can protect against these events. It occurs directly through the control of hypertension and indirectly through other cardiovascular effects. This meta-analysis and systematic review aimed to assess the impact of various antihypertensive medications (ACE inhibitors, beta-blockers, calcium channel blockers, diuretics, etc.) on blood pressure and various cardiovascular outcomes. A thorough search was conducted using several online databases and search engines, including PubMed, Google Scholar, ScienceDirect, Medline, Embase, and others. RCTs evaluating the impact of antihypertensive medications on BP and other cardiovascular events like coronary heart disease and stroke were included in this study. Included were studies detailing the use of antihypertensive medication in monotherapy. The meta-analysis was done using RevMan version 5.4 software (Cochrane Collaboration, London, UK). Means and standard deviations were extracted for the continuous variables and events, and the total sample number was extracted for the dichotomous variables. This analysis encompassed a total of 18 RCTs of the elderly population. The data for each variable was extracted independently, and analysis was performed. Overall, systolic blood pressure (SBP) revealed an impact of -11.88, CI=95% (-20.56, -3.19). The diastolic blood pressure (DBP) showed -5.41, CI=95% (-9.62, -1.20), myocardial infarction 0.92, CI=95% (0.82, 1.04), stroke 0.83, CI=95% (0.74, 0.94), and cardiovascular mortality 0.93, CI=95% (0.86, 1.00). Heterogeneity was present due to the variable sample size of the studies and other unidentified biases. In conclusion, there was a significant reduction in the elderly population's risk of stroke, myocardial infarction, and cardiovascular death when antihypertensive medications were taken.

## Introduction and background

Hypertension is a state of high blood pressure (BP) in which the blood vessels have persistently raised pressure. The normal values range from systolic lower than 120 mmHg to diastolic lower than 80 mmHg. Hypertension is one of the fifth attributable fraction of heart diseases [1]. High BP significantly increases the risk of incidents of cardiovascular disease (CVD) in adults [2-4]. The prevalence of hypertension has dramatically increased over the last 40 years, both globally and in the United States (US), with an increased incidence in the elderly [5]. The prevalence may be as high as 79% and 85% in people aged 65 to 74 and 77% and 75% in people aged 75 to 74, respectively [6]. With 12.9 million deaths and 0.3 billion disability-adjusted life years (DALYs) annually, CVD is the leading cause of death and DALYs worldwide [7,8]. The largest risk factor for CVD is elevated BP, which affected 1.13 billion people worldwide in 2015 [9,10]. The goal and strategy for taking care of hypertension in the elderly are very different than in younger people. Concerns for patients and clinicians usually center on the safety of antihypertensive drugs, polypharmacy, the efficacy of treating hypertension in the elderly, and, most importantly, adverse side effects like orthostatic hypotension, which increase the risk of fractures and falls [11]. According to an observational study, older patients receiving more intensive antihypertensive therapy may have a higher death rate [12]. An important dilemma in treating hypertension in the elderly involves determining this group's ideal BP target. The BP thresholds for treatment in the elderly have been lowered in accordance with American and European guidelines. The target systolic blood pressure (SBP) for elderly patients is 130 mmHg by European standards and 140/90 mmHg in the US American College of Cardiology and the American Heart Association guidelines from 2017 [13]. Previous RCTs have demonstrated the benefits of BP-lowering therapy in preventing cardiovascular events, and numerous meta-analyses have assessed the combined effectiveness of BP-lowering medications in various groups. There was a major reduction of major cardiovascular events by 20% with every 10 mmHg systolic pressure reduction (relative risk (RR) 0.80, 95% confidence interval (CI) 0.77 to 0.83), coronary heart disease by 17% (RR 0.83, 95% CI 0.78 to 0.88), stroke by 27% (RR 0.73, 95% CI 0.68 to 0.77), heart failure by 28% (RR 0.72, 95% CI 0.67 to 0.78), and death from all causes by 13% (RR 0.87, 95% CI 0.84 to 0.91) [14-16]. Secondary prevention of subsequent cardiovascular events among survivors of CVD is essential to lowering the high recurrence rate [17]. While randomized studies have demonstrated that lowering BP in the elderly is beneficial, most of these studies have regularly recruited individuals who weren't weak and were generally in good health, restricting the application of the data demonstrating the efficacy of antihypertensive medication in the overall senior population. The Blood Pressure Systolic SPRINT Intervention Trial is an exception that demonstrates comparable benefits in older people who are not frail [18].

The rationale

A systematic review and meta-analysis are needed to assess the effectiveness and safety of antihypertensive medications in the elderly, considering their high hypertension prevalence and unique response to treatment in this population and subgroup.

Aims and objectives

This meta-analysis and systematic review sought to determine how various antihypertensive medication classes, such as ACE inhibitors, beta-blockers, calcium channel blockers, diuretics, and others, affect BP levels and various cardiovascular events. The comprehensive evaluation of the efficacy of various antihypertensive drug classes in reducing BP and preventing cardiovascular events in the senior population is the goal of this systematic review and meta-analysis. Through a comprehensive analysis of the available data, this study aims to produce evidence-based suggestions that can guide clinical guidelines, policymakers, and healthcare professionals in the best possible way when choosing antihypertensive medications for the management of elderly patients' hypertension. This will ultimately improve the patient's overall health and quality of life over the long term.

## Review

Methodology

Study Design

We conducted a meta-analysis and systematic review for this study. Using the population, intervention, control, and outcome of the PICO framework, a systematic review question was developed.

Eligibility Criteria

The PICOS scheme (population, intervention, comparison, outcome, and study design) was utilized to establish the eligibility requirements for studies in accordance with the recommendations provided by the Preferred Reporting Items for Systematic Reviews and Meta-Analysis (PRISMA). The inclusion criteria for this systematic review and meta-analysis are as follows: (1) Based on their titles and abstracts, studies are judged eligible after full texts have been screened. (2) This systematic review and meta-analysis only contained RCTs. (3) Included were studies detailing the use of antihypertensive medication in monotherapy. (4) Included were studies that reported on outcomes in older adults (above 60 years of age). (5) Every study conducted in English was included. The following are the exclusion standards for this systematic review and meta-analysis: (1) research that wasn't RCT-based, (2) education conducted in a language other than English, and (3) research pertaining to age groups younger than 60.

Search Strategy

The search strategy adopted for this systematic review includes a vast search for the literature on various databases such as PubMed, Google Scholar, and Web of Science. The PRISMA guidelines were followed throughout the search for articles. Different journal titles, abstracts, and full-text articles were found. Boolean operators AND/OR were used for the search strategy. Multiple filters were also implied to make the search for articles specific.

Data Extraction

PRISMA was used to guide the systematic review. A thorough search of various electronic databases, including PubMed, Web of Science, Science Direct, and the Cochrane Library, was conducted as part of the methodology. The studies retrieved included those focusing on the effects of antihypertensive medications on cardiovascular events. The search strategy included the following keywords: (efficacy OR synonyms) AND (antihypertensives OR synonyms) AND (blood pressure OR synonyms) AND (cardiovascular events OR synonyms) AND (elders OR synonyms). There were two phases to the article screening process. The titles and abstracts of every article found in the chosen electronic databases were examined during the first screening phase. From this review, a list of papers was compiled for possible inclusion. The complete texts of the articles that passed the first screening round were obtained for the second screening round. For every eligible paper, uniform data extraction tables with information on the first author, the year the study was published, the study design, the country of study, the location, the setting (rural or urban), the sample size, the sampling technique, the data source, the instrument, standard errors (SEs) and standard deviations (SDs), and related factors were tabulated. Disagreements were settled through discussion and the senior author's judgment.

Selection Process

We searched peer-reviewed journals and publications for relevant literature in order to meet the inclusion criteria. We tried to "include" or "exclude" pertinent studies based on the inclusion and exclusion criteria. In all, 18 studies were taken into account for the final review and analysis. Research that failed the eligibility requirements for screening was classified as "dispute" or "exclusion." Prior to removing a study from the literature, the exclusion criteria were presented. Studies were disqualified for two reasons: (1) the population had an issue; (2) we discovered a high risk of bias.

Statistical Analysis

Data for each variable was extracted manually for meta-analysis. For the dichotomous variable, the total sample size and events were extracted for both the experimental and control groups. For continuous variables, mean, SD, and total sample sizes were extracted again for both experimental and control groups. The original plan for crossover studies was to extract data from paired t-tests, which would have made it possible to assess how each subject's measurements for the intervention and control differed from one another. Sadly, the studies hardly ever included these kinds of data, so a different strategy was used. Crossover studies may find it more difficult to identify true intervention effects as a result of this cautious data extraction strategy. SE was used to measure SD in the primary studies where SD was not provided. Additionally, when data were displayed graphically (e.g., figures), an attempt was made to estimate numerical values for the results. All food or flavonoid groups, as well as any trials with pertinent outcome data, were included in the primary analyses. The presence of heterogeneity, or real variation in effect sizes, was assessed using a P 0.1 threshold. Heterogeneity was used to gauge the degree of the discrepancy between studies; a 50% threshold was considered significant.

Heterogeneity and Reporting Bias

In a meta-analysis, heterogeneity assessment refers to the process of determining whether the differences between the studies that are part of the meta-analysis are significant enough to impact the overall results. To guarantee the accuracy and dependability of the meta-analysis findings, this evaluation is crucial. Apart from the R^2^ statistic, the I^2^ and Tau^2^ statistics can be used to assess heterogeneity in the meta-analysis.

Quality Assessment

In order to evaluate the potential for bias in the studies that were chosen for the meta-analysis, we looked for digital and web resources. The RoB 2 tool (Cochrane Collaboration, London, UK) was used to evaluate seven risk domains that were included in the primary studies. The meta-analysis looked at selection bias caused by random sequence generation, allocation concealment, participant and personal blinding, outcome assessment blinding, attrition bias caused by incomplete outcome data, selective reporting, other biases, and four other risk-of-bias domains [19-20]. There is a traffic light plot in the Results section.

Results

Data Items

**Figure 1:**
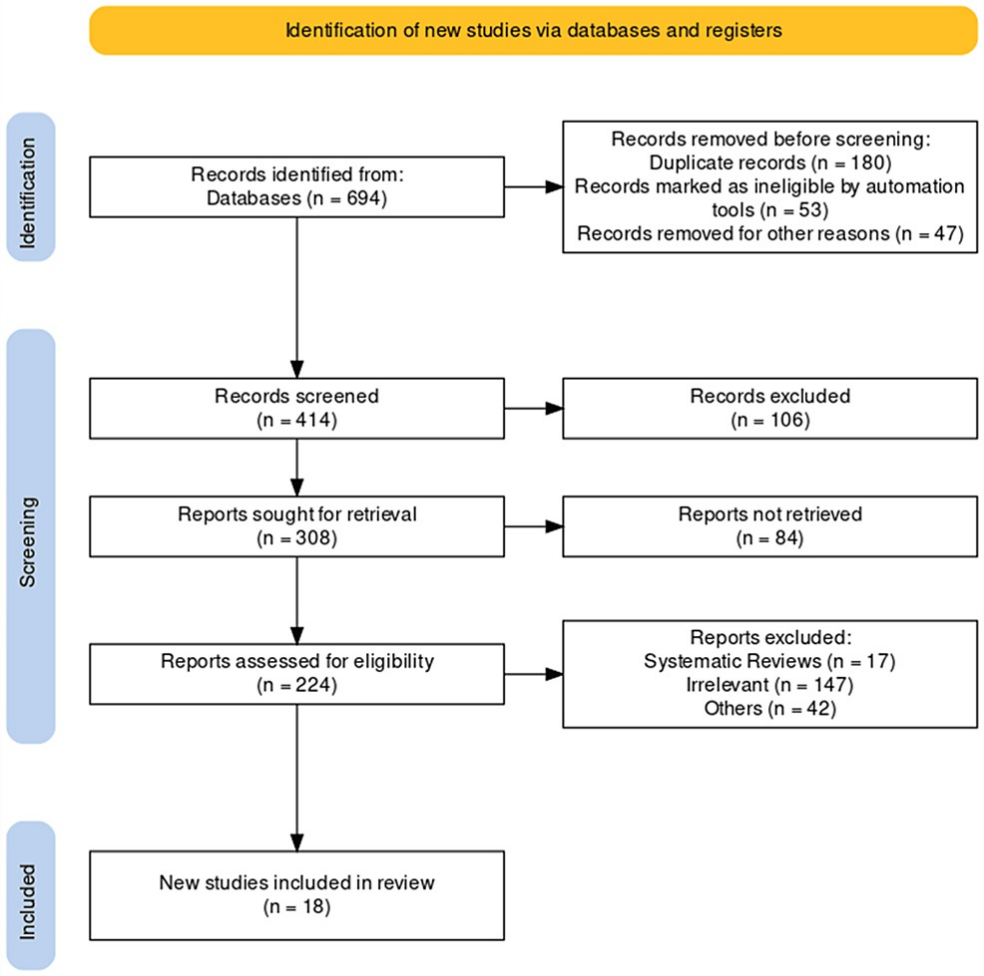
PRISMA flowchart of the included studies

Following the completion of the secondary screening procedure, a thorough examination of the entire sample size (n=18) from the selected literature was carried out. To create a PRISMA flow diagram, the researchers followed the PRISMA guidelines. Figure 1 depicts the flow of the study selection process, which includes the identification, screening, eligibility, and inclusion of studies from journals and other independent resources based on report availability.

We tabulated each study intervention individually after the study selection process was finished. The synthesis table contained only references to the outcome's pertinent themes.

Study Characteristics

There were 18 peer-reviewed studies in the final sample used for systematic analysis. A summary of the study characteristics and demographic information from the included trials can be found in Table 1.

**Table 1:** Study characteristics of all included studies ACE inhibitor: angiotensin-converting enzyme inhibitor, BP: blood pressure [21-38]

Sr no.	Study	Study design	Country	Mean age	Sample size (n)	Drug(s) used	Results
1	Yusuf et al. 2008 [21]	Randomized controlled trial	Canada	66.9	Experimental 2,954, control group 2,972	Telmisartan 80 mg/day	Patients who could not take ACE inhibitors tolerated telmisartan well. The drug did not significantly affect the study's primary outcome, which included heart failure hospitalizations.
2	Trenkwalder et al. 2005 [22]	Randomized controlled trial	Germany	75	Experimental 52,477 or placebo group 52,460	Candesartan 8-16 mg daily	In all patient subgroups, candesartan-based therapy consistently improves major cardiovascular events and stroke. Nonetheless, patients who had previously experienced a stroke at the beginning of the study benefited the most.
3	Lithell et al. 2003 [23]	Randomized double-blind intervention trial	Sweden	76.4	Experimental 2,477, control 2,460	Candesartan 8-16 mg once daily	An elderly hypertensive patient's slightly better BP reduction during candesartan-based therapy was associated with a marked decrease in non-fatal stroke and a modest, statistically nonsignificant reduction in major cardiovascular events when compared to control therapy.
4	Yusuf et al. 2008 [24]	Randomized controlled trial	Canada	66.1	Experimental 10,146, control 10,186	Telmisartan 80 mg/day	The study discovered that using telmisartan in this context did not result in a significant reduction in the occurrence of subsequent strokes, major cardiovascular events, or the onset of diabetes.
5	McMurray et al. 2015 [25]	Randomized controlled trial	40 countries	63.7	Experimental 4,631, control 4675	After two weeks, the dose of valsartan was increased to 160 mg once daily from the initial 80 mg	The use of valsartan in conjunction with lifestyle modification decreased the incidence of diabetes by a relative of 14%; however, it did not affect the rate of cardiovascular events.
6	Massie et al. 2008 [26]	Randomized controlled trial	25 countries	72	Experimental 2,067, control 2,061	75 mg of irbesartan daily	Individuals with heart failure, and left ventricular ejection fraction did not benefit from irbesartan. In other words, the use of irbesartan did not have a beneficial effect on this specific patient population suffering from heart failure.
7	Disertori et al. 2009 [27]	Randomized controlled trial	Different countries	67.5	Experimental 722, control 720	Valsartan	There was no correlation found between valsartan treatment and a lower incidence of recurrent atrial fibrillation.
8	Yusuf et al .2011 [28]	Randomized controlled trial	40 countries	69.5	Experimental 4,518, control 4,498	Irbesartan once daily for two weeks at a dosage of 150 mg	Patients with atrial fibrillation did not see a decrease in cardiovascular events when using irbesartan.
9	Yusuf et al. 2000 [29]	Randomized controlled trial	Canada	66	Experimental 4,645, control 4,652	2.5 mg of ramipril once a day	Ramipril significantly reduces the risk of cardiovascular problems in a large number of high-risk patients without a history of heart failure.
10	Fox et al. 2003 [30]	Randomized controlled trial	United Kingdom	60	Experimental 6,110, control 6,108	4 mg oral perindopril once daily	Perindopril can greatly enhance the prognosis of individuals with stable coronary heart disease who do not appear to be experiencing heart failure.
11	Staessen et al. 1998 [31]	Randomized controlled trial	Belgium	62	Experimental 2,398, control 2,287	Nitrendipine 40 mg/d	It might lessen risk factors in senior citizens.
12	Staessen et al. 1998 [32]	Randomized controlled trial	Belgium	62	Experimental 2,398, control 2,287	Nitrendipine 40 mg/d	When antihypertensive medication treatment with nitrendipine is started, the rate of cardiovascular complications is reduced in elderly patients with isolated systolic hypertension. If 1000 patients receive this kind of regimen for five years, it may be possible to prevent 53 major cardiovascular endpoints or 29 strokes.
13	Liu et al. 1998 [33]	Randomized controlled trial	China	66.7	Experimental 1,253, control 1,141	Nifedipine	Elderly Chinese patients with hypertension benefit from antihypertensive medication treatment in preventing stroke and other cardiovascular complications.
14	Wilson et al. 2004 [34]	Randomized controlled trial	United Kingdom	63.5	Experimental 3,825, control 3,840	Nifedipine	If nifedipine is added to the conventional treatment for angina pectoris, there is no effect on major cardiovascular event-free survival. It is less necessary to undergo coronary angiography and intervention when using nifedipine.
15	Black et al. 1991 [35]	Randomized controlled trial	Different countries	71.6	Experimental 2,365, control 2,371	Chlorthalidone	The use of chlorthalidone decreases the incidence of stroke in patients older than 60 years.
16	Peart et al. 1992 [36]	Randomized controlled trial	United Kingdom	70.2	Experimental 773, control 738	Hydrochlorothiazide and amiloride	Amiloride and hydrochlorothiazide reduce the incidence of cardiovascular problems in older adults with hypertension.
17	Bulpitt et al. 2003 [37]	Randomized controlled trial	United Kingdom	83.6	Experimental 386, control 394	Diuretics and ACE inhibitors	The main trial should continue as planned, according to the preliminary results. A year's worth of treatment for 1000 patients may result in an additional 20 non-stroke deaths in addition to a 19% reduction in stroke events (nine non-fatal).
18	Beckett et al. 2008 [38]	Randomized controlled trial	United Kingdom	83.6	Experimental 1,933, control 1,912	Perindopril	The findings support the benefits of antihypertensive therapy for people 80 years of age and above, whether it involves perindopril or indapamide (sustained release).

Meta-analysis

Data from multiple research studies was gathered for this investigation in order to conduct a systematic review or meta-analysis. The number of participants in each study arm was specifically documented for continuous outcomes measured on a continuous scale. The means and SDs of the changes in outcome variables measured from the beginning to the end of the intervention period were extracted separately for both the intervention and control groups in studies where interventions were conducted. When these specific data were unavailable, the means and standard deviations of the intervention and control groups' outcome measurements were used as a substitute. Every trial with relevant outcome data was included, and primary analyses were performed for each score. The meta-analysis was carried out using RevMan version 5.4 software (The Cochrane Collaboration, London, UK).

Systolic Blood Pressure

**Figure 2:**
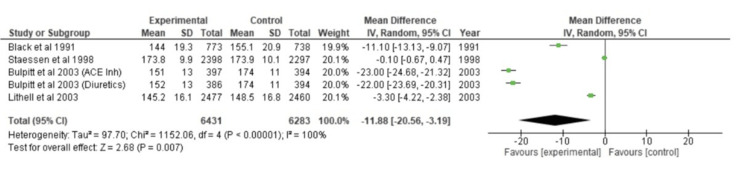
Forest plot of SBP CI: confidence interval, ACE inh: angiotensin-converting enzyme inhibitor, SBP: systolic blood pressure [23,32,35,37]

For every one of the five different studies, a forest plot was made for continuous data. To calculate the deviation and changes in mean (m) and SD using the SMD scale, a random-effects model was chosen. The horizontal axis was used to calculate the CI (95%), and the plot's "point estimation" was shown as green squares. The vertical line in the center denotes a condition of "no effect." After calculating the overall effect size using Cohen's d, the result was d=-11.88, CI=95% (-20.56, -3.19). According to the calculations, the heterogeneity was Tau^2^=97.70, Chi^2^=1152.06, df=3 (p<0.00001), and I^2^=100%. After analysis, Z=2.68 (p=0.007) was determined to be the overall effect. Every study's individual effects were in favor of the experimental group. The forest plot has been shown in Figure 2.

Diastolic Blood Pressure

**Figure 3:**
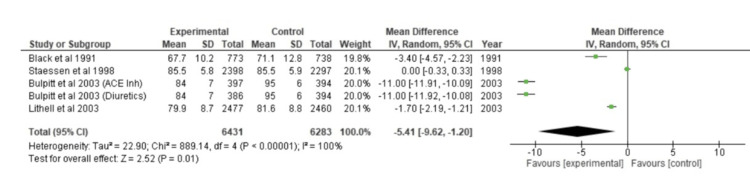
Forest plot of DBP CI: confidence interval, ACE inh: angiotensin-converting enzyme inhibitor, DBP: diastolic blood pressure [23,32,35,37]

The forest plot was made for five different studies using continuous data. When Cohen's d was used to calculate the overall effect size, the outcome was d=-5.41, CI=95% (-9.62, -1.20). According to the calculations, the heterogeneity is as follows: I^2^=100%; Tau^2^=22.90; Chi^2^=889.14, df=4 (p<0.00001). Z=2.52 (p=0.01), the analysis for the overall effect, was discovered. Every study's individual effects were in favor of the experimental group. The forest plot has been shown in Figure 3.

Myocardial Infarction

**Figure 4:**
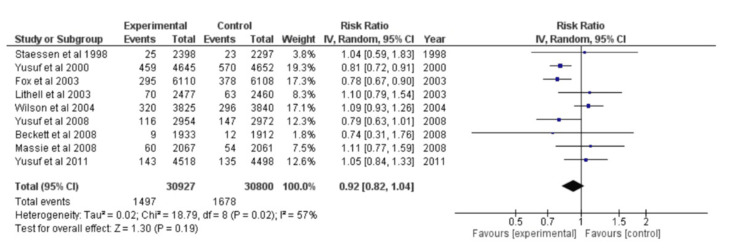
Forest plot of myocardial infarction CI: confidence interval [23,24,26,28-31,34,38]

Once more, the forest plot for nine distinct studies was created, but this time it was done for binary data. A model with random effects was chosen. After calculating the overall effect size using Cohen's d, the result was d=0.92, CI=95% (0.82, 1.04). According to the calculations, the heterogeneity was Tau^2^=0.02, Chi^2^=18.79, df=8 (p=0.02), and I^2^=57%. Z=1.30 (p=0.19) for the overall effect was the result of the analysis. Every study's individual effects were different. The forest plot has been shown in Figure 4.

Stroke

**Figure 5:**
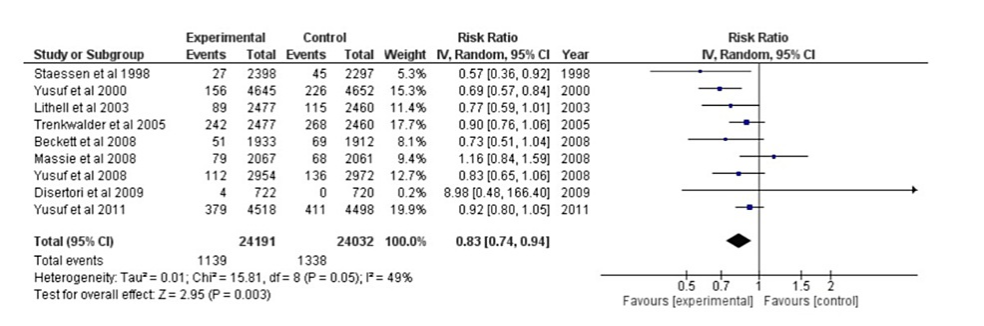
Forest plot of stroke CI: confidence interval [22-24,26-29,32,38]

Dichotomous data was plotted using the forest plot method for nine distinct studies. A model with random effects was chosen. After calculating the overall effect size using Cohen's d, the result was d=0.83, CI=95% (0.74, 0.94). The following values were calculated for the heterogeneity: I^2^=49%; Tau^2^=0.01; Chi^2^=15.81, df=8 (p=0.05). Z=2.95 (p=0.003), the analysis for the overall effect, was discovered. Every study's individual effects were in favor of the experimental group. The forest plot is shown in Figure 5.

Cardiovascular Mortality

**Figure 6:**
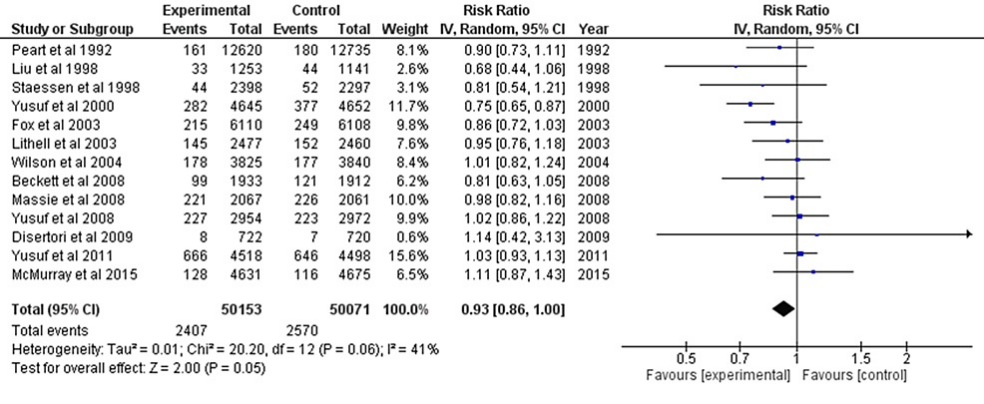
Forest plot of cardiovascular mortality CI: confidence interval [23-30,32-34,36,38]

For dichotomous data, the forest plot for 13 distinct studies was created. A model with random effects was chosen. After calculating the overall effect size using Cohen's d, the result was d=0.93, CI=95% (0.86, 1.00). According to the calculations, the heterogeneity was Tau^2^=0.01, Chi^2^=20.20, df=12 (p=0.06), and I^2^=41%. After analysis, Z=2.00 (p=0.05) was determined to be the overall effect. Every study's individual effects were in favor of the experimental group. The forest plot is shown in Figure 6.

Risk-of-Bias Assessment

**Figure 7:**
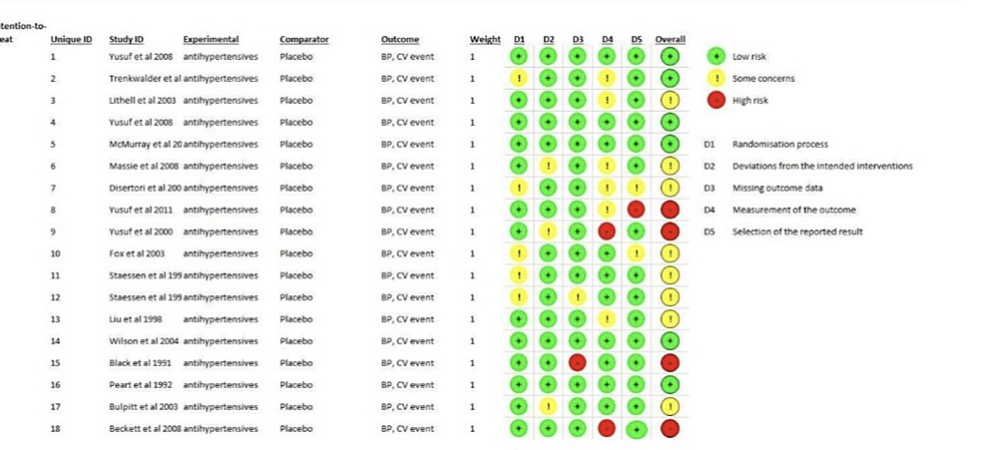
Traffic light plot of individual studies BP: blood pressure, CV event: cardiovascular event

**Figure 8:**
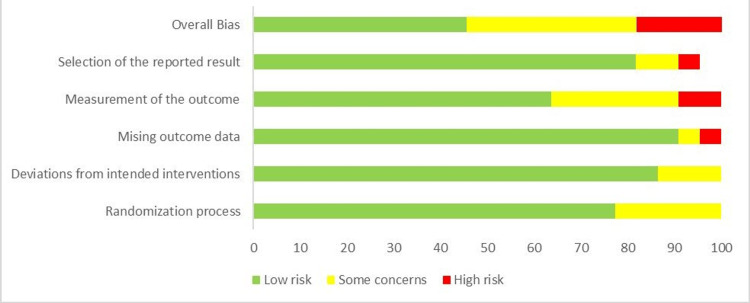
Summary of risk of bias

RoB 2 was used in the study to assess the risk of bias in each primary study chosen for meta-analysis. The final analysis included only studies with a "low" overall bias risk. The researchers used RoB 2 to generate a visual representation known as a "traffic lights" plot to assess and display the bias risk in 18 selected studies, as shown in Figures 7-8.

This evaluation of the efficacy of antihypertensive drugs on BP and cardiovascular events was studied in the elderly population. This showed that almost all antihypertensives were effective in lowering BP (both SBP and DBP) in the elderly population. Using antihypertensive drugs in the elderly population significantly reduced the incidence of stroke, myocardial infarction, and cardiovascular mortality. The meta-analysis offered here supplements the results previously published [39] to address the commonly asked question of whether older and younger people should receive different hypertension treatments. Even so, prior research revealed that comparable RR reductions in all cardiovascular events, both fatal and nonfatal, can be attained by lowering BP in people, both over 65 and under 65 (and absolute risk reductions are greater in elderly patients), and that obtaining these goals leads to gradual drops in cardiovascular risk SBP/DBP significantly lower than 140/80 mmHg without a clear increase in treatment withdrawals due to unfavorable outcomes.

The current meta-analysis has examined whether various BP-lowering drug classes have a preferred impact on the elderly population. In another systematic review, Thomopoulos et al.'s research indicates that, for treating hypertension, antihypertensive drugs effectively lower the risk of nonfatal cardiovascular events in both younger and older individuals. However, while beta-blockers are as effective as other drug classes in people under 65, they are less effective in older individuals, with atenolol being particularly less effective in the elderly. The causes of this decreased efficacy with aging have not been investigated in the majority of trials, so they cannot be examined using meta-analyses [39]. Individuals aged 65 and older, as well as those aged 75 and older, who received antihypertensive therapy, had a significantly lower risk of all-cause and cardiovascular mortality, heart failure, and stroke, according to Murad et al. This benefit was consistent regardless of whether or not the individuals had diabetes [40]. Xie et al. showed that ACE inhibitors considerably reduced the risk of stroke, myocardial infarction, cardiovascular events, and all-cause mortality. Head-to-head comparisons, however, did not yield compelling proof that the effects of these BP-lowering medications differed [41]. Ettehad et al.'s meta-analysis measured how BP-lowering medications affected cardiovascular outcomes [42]. It was discovered that calcium channel blockers outperformed other medications, and beta-blockers were less effective than other medications for the prevention of stroke.

Several limitations were present in our study. Firstly, only antihypertensive drugs were targeted without comparing different classes, potentially restricting the applicability of our findings to the overall effect. Furthermore, despite the absence of substantial indications for publication bias and the other effects observed from different classes, significant heterogeneity emerged within the selected studies. This significant heterogeneity might arise from differences in study designs and application protocols employed across the included studies.

## Conclusions

This research evaluated the efficacy of antihypertensive medications on BP and cardiovascular events in the elderly population. This study demonstrated the efficacy of nearly all antihypertensives in lowering SBP and DBP in the elderly. The risk of cardiovascular events in the elderly population was reduced significantly when treated with antihypertensive drugs.

## Appendices

This meta-analysis and systematic review titled “The Efficacy of Antihypertensive Drugs in Lowering Blood Pressure and Cardiovascular Events in the Elderly Population: A Systematic Review and Meta-Analysis." Here are a few potential research questions that could guide the study:

1. Primary research question:

How do antihypertensive medications (ACE inhibitors, beta-blockers, calcium channel blockers, diuretics, etc.) impact systolic and diastolic blood pressure in the elderly?

2. Secondary research questions:

How do different classes of antihypertensive medications compare in their effectiveness in reducing systolic and diastolic blood pressure?

What is the effect of antihypertensive medication monotherapy on the incidence of myocardial infarction in the elderly?

Does antihypertensive medication reduce the risk of stroke in the elderly population? If so, how significant is this reduction?

How do antihypertensive medications influence cardiovascular mortality rates in the elderly?

Is there a variation in the effectiveness of antihypertensive medications based on the sample size and characteristics of different studies?

3. Methodological research question

What is the level of heterogeneity among the studies included in the meta-analysis, and what are its potential sources?
